# The low abundance of clonally expanded mitochondrial DNA point mutations in aged substantia nigra neurons

**DOI:** 10.1111/j.1474-9726.2009.00492.x

**Published:** 2009-08

**Authors:** Amy K Reeve, Kim J Krishnan, Geoffrey Taylor, Joanna L Elson, Andreas Bender, Robert W Taylor, Christopher M Morris, Doug M Turnbull

**Affiliations:** 1 Mitochondrial Research Group, Newcastle University Centre for Brain Ageing and Vitality, Institute for Ageing and Health, Newcastle UniversityNewcastle upon Tyne, NE2 4HH, UK; 2 Department of Neurology, University of Munich, Klinikum GrosshadernMarchioninistr. 15, 81377 Munich, Germany; 3 Medical Toxicology Centre, Institute of Neuroscience, Newcastle UniversityNE1 7RU, UK; 4 Institute for Ageing and Health, Newcastle UniversityNewcastle upon Tyne, NE4 6BE, UK

**Keywords:** clonal expansion, mtDNA, neurons, point mutations

## Abstract

Clonally expanded mitochondrial DNA (mtDNA) deletions accumulate with age in human substantia nigra (SN) and high levels cause respiratory chain deficiency. In other human tissues, mtDNA point mutations clonally expand with age. Here, the abundance of mtDNA point mutations within single SN neurons from aged controls was investigated. From 31 single cytochrome *c* oxidase normal SN neurons, only one clonally expanded mtDNA point mutation was identified, suggesting in these neurons mtDNA point mutations occur rarely, whereas mtDNA deletions are frequently observed. This contrasts observations in mitotic tissues and suggests that different forms of mtDNA maintenance may exist in these two cell types.

The mitochondrial genome is present in multiple copies, the number varying depending on the energy requirement of the cell. Mitochondrial DNA encodes key respiratory chain proteins, as well as RNA machinery required for intra-mitochondrial protein synthesis (Anderson *et al.*, 1981). Mitochondrial DNA defects are an important cause of mitochondrial disease with an incidence of ∼1 in 10 000 adults clinically affected in the UK (Schaefer *et al.*, 2008). Although patient symptoms are variable, severe and progressive neurological problems are a common feature (Taylor & Turnbull, 2005).

Mitochondrial DNA mutations accumulate with age in many tissues from human subjects (Liu *et al.*, 1998; Fayet *et al.*, 2002; Nekhaeva *et al.*, 2002; Taylor *et al.*, 2003), although their contribution to the human aging process remains uncertain. Both mtDNA point mutations and large-scale deletions clonally expand to high levels (> 60%) leading to respiratory chain deficiency. Support for a direct role for mtDNA mutations in aging resulted from the generation of mouse models with a proof-reading deficient version of PolgA; these mice have high levels of mtDNA mutations and develop a premature aging phenotype (Trifunovic *et al.*, 2004; Kujoth *et al.*, 2005; Vermulst *et al.*, 2008).

Although both mtDNA deletions and point mutations are observed in human cells, few studies have examined the presence of both in the same tissue. This may give insight into the mechanisms by which they arise and clonally expand. We previously detected high levels of mtDNA deletions in substantia nigra (SN) neurons from aged humans (Bender *et al.*, 2006; Krishnan *et al.*, 2008; Reeve *et al.*, 2008). In this study, we wished to determine if these cells also contained clonally expanded mtDNA point mutations.

Frozen midbrain sections were cut at 20 μm from five elderly controls (Table 1). Mitochondrial dysfunction was determined using sequential histochemical staining for the activities of cytochrome *c* oxidase (COX) and succinate dehydrogenase (Brierley *et al.*, 1998). This revealed the presence of both COX normal and deficient SN neurons. Single COX normal cells were chosen for analysis to allow comparison with mtDNA deletion levels from our previous study (Bender *et al.*, 2006).

**Table 1:** Table showing the details of the subjects used in this study

Subject number	Age	pm delay (h)	Sex	mtDNA haplogroup	Haplogroup markers	Percentage COX-deficient neurons in the SN (Bender *et al.*, 2006)	Percentage deletion from 25 COX normal neurons (Bender *et al.*, 2006)
1	75	30	M	K	m.3480A>G, m.9698T>C, m.10550A>G, m.11299T>C	0.61	40
2	72	28	F	J	m.11251A>G, m.12612A>G, m.13708G>A, m.15452C>A	0.35	45
3	75	9	F	H2	m.1438A>G, m.4769A>G, m.8860A>G, m.15326A>G	0.26	42
4	81	29	F	H2	m.1438A>G, m.4769A>G, m.8860A>G, m.15326A>G	0.12	33
5	89	27	M	X	m.6221T>C, m.6371C>T, m.13996A>G, m.14470T>C	0.42	56

pm, post mortem; COX, cytochrome *c* oxidase; SN, substantia nigra.

The entire mtDNA was sequenced from 31 single SN neurons as previously described (Taylor *et al.*, 2001). Analysis of single cell mtDNA sequences is problematic as highlighted previously (Bandelt *et al.*, 2008). Low mtDNA copy number in single cells can result in contamination from nuclear pseudogenes or result in PCR-induced errors. To ensure detection of only true clonally expanded mutations, we re-sequenced all putative mutations with DNA from the initial cell lysate; repeating the second-round PCR, to confirm the presence of a mutation by at least two, separate PCR reactions. Following re-sequencing, we excluded 10 mutations detected only on the initial PCR reaction. None were recognized polymorphisms, suggesting the mutations arose during PCR amplification.

In total, we analysed 31 cells (513 639 bp). Germline polymorphisms were found in all cells from each subject. All changes from the revised Cambridge Reference Sequence (Andrews *et al.*, 1999) (*n* = 730) were recognized polymorphic variants (MITOMAP, 2009, Ingman & Gyllensten, 2006), permitting the determination of mitochondrial haplogroups, for all subjects (Table 1).

True clonally expanded point mutations were those present only once in a single cell from an individual. Using these criteria, we detected only one clonally expanded point mutation (Table 1, Fig. 1A). This m.5480A>C transversion found within the MTND2 gene, NADH ubiquinone oxidoreductase (complex I) subunit 2, was synonymous and was heteroplasmic (∼30%) based on the relative abundance of peaks in the sequence chromatogram. This shows that clonal expansion of mtDNA point mutations is a rare event in single SN neurons, consistent with previous data from a small number of COX-deficient cells (Bender *et al.*, 2006). The single clonally expanded point mutation detected does not confer an amino acid change and is therefore unlikely to be pathogenic. Based on these data, only 3% (1/31) of SN neurons from the elderly subjects harbour a clonally expanded point mutation, a very different scenario to mtDNA deletions, where high levels of deletions were detected in COX normal neurons from the same subjects (Table 1) and other elderly subjects (Bender *et al.*, 2006; Kraytsberg *et al.*, 2006).

**Fig. 1:**
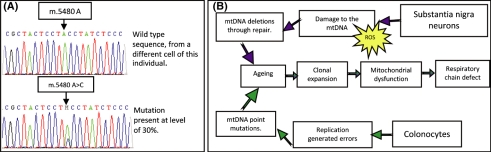
(A) Sequence electropherogram of the clonally expanded point mutation detected within the SN neurons. (B) Flow diagram to illustrate how different mtDNA mutations may be preferentially formed in mitotic and postmitotic tissues. Both mutation types lead to mitochondrial dysfunction with age. ROS = Reactive oxygen species.

Both mitotic and postmitotic cells show clonal expansion of mtDNA mutations with age, but it appears that there are marked differences in the type of mutations detected. In buccal epithelium cells and cardiomyocytes, clonally expanded mtDNA point mutations have been documented; however, mtDNA deletions were only detected in the cardiomyocytes (Bodyak *et al.*, 2001; Nekhaeva *et al.*, 2002). In a separate study, in three different tissues (skeletal muscle, heart and kidney), the 4977 bp mtDNA deletion and an m.3243 A>G point mutation accumulated with age. However, deletion levels were much higher in skeletal muscle and point mutation accumulation occurred earlier in the kidney and heart reaching higher levels than in skeletal muscle (Liu *et al.*, 1998). These studies imply that there is preferential formation and/or accumulation of mtDNA point mutations in mitotic tissues, whereas, mtDNA deletions accumulate in postmitotic tissues (Fig. 1B).

Mitochondrial DNA replication occurs in postmitotic cells, even though the cell cycle is suspended. The rate of mtDNA turnover remains unknown, but it is thought to be much slower in neurons than in dividing cells (Wang *et al.*, 1997). Substantia nigra neurons are particularly vulnerable to reactive oxygen species damage, due in part to their high iron content and through dopamine metabolism (Halliwell, 1992). Thus the high levels of mtDNA deletions are most likely to be caused during repair of the reactive oxygen species damage (Krishnan *et al.*, 2008). In mitotic tissues where cell turnover occurs more frequently, mtDNA mutations are more likely to be generated by replication errors (Fig. 1B).

In conclusion, we have described low levels of clonally expanded point mutations in SN neurons, which contrast the high levels of mtDNA deletions previously reported. This further supports the difference in mtDNA mutation types detected between mitotic and postmitotic cells, and implies that different mechanisms of mtDNA maintenance exist within different cell types.
